# Toxicity Associated with Pembrolizumab Monotherapy in Patients with Gastrointestinal Cancers: A Systematic Review of Clinical Trials

**DOI:** 10.3390/biomedicines13010229

**Published:** 2025-01-18

**Authors:** Nikolas Naleid, Amit Mahipal, Sakti Chakrabarti

**Affiliations:** 1 Department of Medicine, University Hospitals of Cleveland, Lakeside Building, 11100 Euclid Avenue, Cleveland, OH 44016, USA; 2 University Hospitals Seidman Cancer Center, Case Western Reserve University, Cleveland, OH 44106, USA

**Keywords:** pembrolizumab, safety, programmed cell death receptor 1, toxicity

## Abstract

**Background/Objectives:** Pembrolizumab, an immune checkpoint inhibitor targeting programmed death 1 (PD-1), is a widely employed therapy for various gastrointestinal (GI) cancers. We conducted a systematic review of clinical trials investigating pembrolizumab monotherapy in GI cancer patients to assess the spectrum and incidence of immune-related adverse events (irAEs) associated with pembrolizumab. **Methods:** A comprehensive search of PubMed/MEDLINE was performed to identify clinical trials investigating pembrolizumab monotherapy in GI cancer patients. Primary endpoints included the incidence of grade 3 or higher irAEs and the rate of treatment discontinuation due to irAEs. Secondary endpoints encompassed the incidence of any-grade irAEs, as well as specific irAEs. **Results:** Data extraction and analysis were performed on 25 articles. The analysis included 3101 patients with a median age of 62 years (range 53–68), with 30.2% being female. Tumor types encompassed were colorectal (12%), esophagogastric (46%), hepatocellular carcinoma (24%), and other GI tumor types (18%). The rate of treatment discontinuation due to irAEs was 6.8%. The most prevalent grade 3 or higher irAEs were hepatitis (3.6%), pneumonitis (0.8%), and colitis (0.7%). Death attributed to irAEs was infrequent (0.9%). **Conclusions:** In patients with GI cancers treated with pembrolizumab monotherapy, severe toxicities are infrequent, and irAEs leading to treatment discontinuation or death are uncommon.

## 1. Introduction

Immune checkpoint inhibitors (ICIs) [1] represent a breakthrough in cancer immunotherapy, harnessing the body’s immune system to recognize and attack tumor cells. ICIs target regulatory pathways in the immune system, particularly checkpoint molecules that act as brakes to prevent the overactivation of immune responses. Tumors often exploit these checkpoints, such as programmed cell death protein-1 (PD-1), its ligand (PD-L1), and cytotoxic T-lymphocyte-associated protein-4 (CTLA-4), to evade immune detection [1]. ICIs work by blocking these interactions, thereby restoring T-cell activity and enhancing anti-tumor immune responses. PD-1 and PD-L1 inhibitors, including pembrolizumab and nivolumab, are primarily effective in tumors expressing high levels of PD-L1 or exhibiting mismatch repair deficiency and microsatellite instability [1]. CTLA-4 inhibitors, such as ipilimumab, target early T-cell priming and activation, complementing the downstream effects of PD-1 blockade [1]. These agents have revolutionized the treatment landscape for cancers such as melanoma, lung cancer, and gastrointestinal malignancies. ICIs are categorized based on their targets, with PD-1/PD-L1 and CTLA-4 inhibitors being the most widely used. PD-1/PD-L1 inhibitors primarily act within the tumor microenvironment to restore T-cell effector functions, while CTLA-4 inhibitors enhance the activation of naïve T-cells in lymphoid tissues. The combination of these pathways has shown synergistic effects in some cancers but is also associated with an increased risk of immune-related adverse events (irAEs), including pneumonitis, colitis, and endocrinopathies [1]. Despite these challenges, ICIs have demonstrated durable responses in many cancers, leading to prolonged survival.

Pembrolizumab, an immune checkpoint inhibitor (ICI) targeting the programmed cell death receptor 1 (PD-1), has emerged as a pivotal therapeutic agent across various malignancies, including gastrointestinal (GI) cancers [2]. Over the past decade, the anti-tumor efficacy of pembrolizumab has been supported by robust clinical evidence, leading to multiple approvals by the U.S. Food and Drug Administration (FDA) for the treatment of GI cancers, either as monotherapy or in combination with chemotherapy [3,4,5,6,7,8,9,10,11,12,13,14]. Specifically, pembrolizumab has shown significant anti-tumor efficacy in advanced gastroesophageal [7,14] and colorectal (CRC) cancers [3,5,8,9], with approvals based on robust positive outcomes from pivotal phase III randomized clinical trials. These trials demonstrated significant improvements in progression-free survival (PFS), overall survival (OS), and durable response rates in select GI cancer patient populations, particularly those with mismatch repair-deficient (dMMR) or microsatellite instability-high (MSI-H) tumors.

Despite its therapeutic promise, pembrolizumab, like other ICIs, is associated with a distinctive spectrum of adverse events known as immune-related adverse events (irAEs) [15]. These toxicities stem from immune system activation, which, while targeting cancer cells, may also result in unintended inflammation and damage to normal tissues. IrAEs can affect multiple organ systems and manifest at any point during treatment or even after therapy discontinuation. Commonly reported irAEs associated with pembrolizumab include endocrine disturbances (e.g., hypothyroidism, hyperthyroidism, and adrenal insufficiency), pneumonitis, enterocolitis, dermatologic reactions (e.g., rash and pruritus), hepatotoxicity, and nephritis [16]. The severity of these events varies, with some requiring prompt recognition and management, including immunosuppressive therapies, to mitigate potential complications. As such, understanding the safety profile of pembrolizumab is essential for optimizing its use in clinical practice.

Despite the growing body of evidence supporting pembrolizumab’s efficacy and safety in various cancer types, safety data specific to GI cancer patients remain limited. The majority of existing safety data for pembrolizumab are derived from clinical trials and real-world studies involving patients with non-GI cancers, such as non-small-cell lung cancer (NSCLC) and melanoma [16]. The findings from these studies may not be fully generalizable to patients with GI cancers, given the distinct tumor biology, tumor microenvironment, and potential differences in baseline characteristics of this patient population. An additional area of uncertainty pertains to whether pembrolizumab induces unique or atypical adverse events in patients with GI cancers compared to other malignancies. This question remains largely unanswered due to the paucity of GI cancer-specific safety data. Given the increasing use of pembrolizumab in clinical practice, particularly for patients with advanced or refractory GI cancers, addressing this knowledge gap is of paramount importance. A more comprehensive understanding of pembrolizumab-associated adverse events in GI cancers will not only inform treatment decision-making but also guide the development of tailored monitoring and management strategies for this patient cohort. Furthermore, most systematic reviews and meta-analyses on the safety of pembrolizumab published thus far have included patients who received chemotherapy or targeted agents along with pembrolizumab, potentially confounding the attribution of specific adverse events to pembrolizumab itself [17,18,19,20,21,22]. As a result, there is limited large-scale evidence on the safety of pembrolizumab when used as a monotherapy in GI cancer patients.

In light of these considerations, we undertook a systematic review of clinical trials investigating the safety profile of pembrolizumab monotherapy in a population with diverse GI cancers. Our primary objective was to characterize the spectrum and incidence of adverse events associated with pembrolizumab in this specific patient population. By focusing exclusively on monotherapy, we aimed to eliminate the potential confounding effects of combination regimens and provide a clearer picture of the safety profile of pembrolizumab in GI cancer patients. The findings of this review aim to bridge existing knowledge gaps, offering valuable insights for clinicians and researchers while paving the way for future studies to refine the use of pembrolizumab in GI oncology.

## 2. Methods

### 2.1. Search Strategy and Study Selection

We conducted the current systematic review following the Preferred Reporting Items for Systematic Reviews and Meta-Analyses (PRISMA) guidelines to ensure a rigorous and transparent methodology. This systematic review was registered with the Open Science Framework (OSF) on 4 August 2024. The registration can be accessed at https://doi.org/10.17605/OSF.IO/WJ9E4. A comprehensive literature search was performed on 24 April 2024, using PubMed/MEDLINE to identify relevant full-text articles. The detailed search terms and strategies employed in the review are provided in Supplementary Materials Table S1 for reproducibility. Two investigators (SC and NN) independently reviewed the titles and abstracts of all citations generated from the search to identify articles that met the predefined inclusion criteria.

### 2.2. Eligibility Criteria and End Points

The inclusion criteria for this systematic review were defined to ensure the selection of studies directly addressing the research objectives. Eligible studies were required to meet the following requirements: (1) clinical trials published as full-text articles in English language journals and indexed in PubMed/MEDLINE; (2) inclusion of patients with GI malignancies; (3) administration of pembrolizumab as a monotherapy to the study participants; and (4) reporting of adverse events (AEs), with a particular focus on immune-related adverse events (irAEs).

The primary endpoints of interest were the incidence of grade 3 or higher irAEs and the rate of treatment discontinuation attributed to irAEs. Secondary endpoints included the incidence of any-grade irAEs and the occurrence of specific irAEs. These specific adverse events comprised pneumonitis, colitis, hepatitis, myositis, myocarditis, nephritis, pancreatitis, peripheral neuropathy, skin toxicity, endocrine toxicity, infusion reactions, and treatment-related deaths.

This structured approach allowed for a comprehensive evaluation of the safety profile of pembrolizumab monotherapy in patients with GI cancers, focusing on the severity, frequency, and nature of AEs. The detailed endpoint criteria enabled an in-depth analysis of both broad and specific toxicities, contributing to a nuanced understanding of pembrolizumab’s safety in this population.

### 2.3. Data Extraction, Data Synthesis, and Analysis

During the initial study planning phase, a structured Excel datasheet was developed to systematically capture all relevant data elements required for the analysis. This datasheet was designed to facilitate consistent and comprehensive data collection across all included studies. After identifying studies that met the predefined inclusion criteria, one investigator (NN) thoroughly reviewed the selected studies and extracted relevant data, populating the datasheet accordingly. To ensure the reliability and accuracy of the extracted data, a second investigator (SC) independently reviewed the completed datasheet. Any discrepancies identified during this quality check were resolved collaboratively.

### 2.4. Statistical Analysis

The aggregated data were systematically synthesized and presented narratively to offer a comprehensive and cohesive summary of the key findings. This approach was employed to ensure clarity and facilitate the integration of complex datasets into a unified interpretation. Where quantitative analysis was applicable, statistical methods were utilized to support the robustness of the findings. Specifically, the chi-squared test was employed to assess differences in the incidence of grade 3 and 4 adverse events between comparison groups. This test was selected due to its suitability for evaluating categorical data and its ability to detect statistically significant variations in adverse event rates across groups. The results of the chi-squared analysis were reported with corresponding *p*-values to highlight the strength and significance of the observed associations. Where relevant, additional descriptive statistics were included to contextualize and complement the narrative synthesis.

## 3. Results

### 3.1. Selected Studies

**Figure 1:**
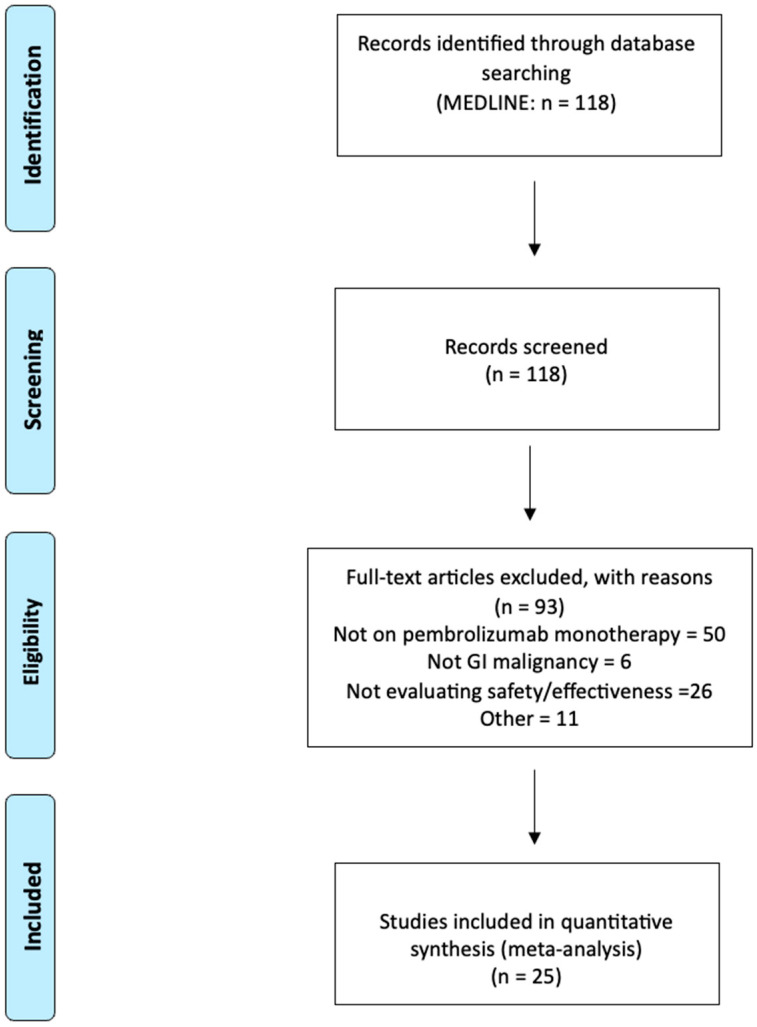
PRISMA flow chart outlining the search, screening, and study selection process.

A total of 118 citations were identified by the search (Figure 1). Following the exclusion of duplicates and irrelevant articles, data extraction and analysis were performed on the 25 selected [3,8,9,10,12,23,24,25,26,27,28,29,30,31,32,33,34,35,36,37,38,39,40,41,42]. The selected studies included 6 phase I studies, 12 phase II studies, and 7 phase III studies. Common reasons for exclusion were the use of concomitant chemotherapy or targeted therapy with pembrolizumab and trials involving non-GI cancer patients. The selected studies are summarized in Table 1.

#### 3.1.1. Patient and Tumor Characteristics

The analysis included data from 3101 patients, with a median age of 62 years (range, 53–68). Among the analyzed cohort, 30.2% of patients were female. Tumor types included colorectal cancer (n = 359, 12%), esophagogastric cancer (n = 1435, 46%), hepatocellular carcinoma (n = 732, 24%), and other gastrointestinal tumor types (n = 575, 18%). This diverse representation of GI malignancies provided a comprehensive dataset for evaluating the toxicity of pembrolizumab monotherapy. Detailed patient demographics and tumor characteristics, including age, gender distribution, and tumor type breakdown, are summarized in Table 2.

#### 3.1.2. Safety

The analysis revealed an overall incidence of grade 3 or higher irAEs of 9.06% (281/3099), with 6.6% (205/3099) of patients discontinuing treatment due to irAEs. The most frequently reported irAEs of any grade included fatigue (497/3099, 16.04%), diarrhea (341/3099, 11%), and hypothyroidism (279/3099, 9%). Among grade 3 or higher irAEs, the most prevalent were pneumonitis (28/3099, 0.9%), hepatitis (27/3099, 0.87%), and colitis (21/3099, 0.68%). Infusion reactions were rare, occurring in only 0.9% of patients, with none reported as grade 3 or higher.

Patients receiving pembrolizumab at a dose of 10 mg/kg every two weeks experienced a significantly higher incidence of grade 3 or 4 adverse events compared to those treated with 200 mg every three weeks (21%, 40/192 vs. 8.4%, 244/2907; *p* < 0.01). Deaths attributed to irAEs were infrequent, occurring in 0.87% of patients (27/3099). A detailed summary of the incidence and types of irAEs is provided in Table 3.

## 4. Discussion

The present systematic review represents the first comprehensive analysis of clinical trial data focused exclusively on the safety profile of pembrolizumab monotherapy in patients with GI malignancies. This analysis highlights pembrolizumab’s favorable safety profile in this specific patient population, characterized by a low incidence of grade 3 or higher irAEs at approximately 9%; a treatment discontinuation rate due to irAEs of around 6%; and a rare incidence of treatment-related mortality, observed in less than 1% of patients. These findings are consistent with the known safety profile of pembrolizumab in broader cancer populations and underscore its safety in clinical practice. Importantly, our analysis did not identify any novel safety signals unique to GI cancer patients, suggesting that the mechanisms underlying pembrolizumab-associated toxicities are consistent across different tumor types. This consistency reinforces pembrolizumab’s utility as a therapeutic option in GI cancers, particularly for patients with biomarkers such as mismatch repair deficiency or microsatellite instability-high status, who derive robust benefits. While the results are encouraging, further studies with larger patient populations and extended follow-up periods are necessary to evaluate long-term safety outcomes, assess late-onset irAEs, and refine patient selection criteria to optimize treatment strategies.

The low incidence of irAEs observed with pembrolizumab monotherapy in this study aligns with our current understanding of immune system regulation and autoimmunity. Under normal physiological conditions, immune responses are regulated by a dynamic balance of co-stimulatory and co-inhibitory signals mediated by immune checkpoint molecules, including PD-1 and PD-L1 [43]. These mechanisms ensure immune tolerance to self-antigens through a dynamic balance of co-stimulatory and co-inhibitory interactions [43]. Cytotoxic T lymphocyte-associated antigen 4 (CTLA-4) inhibitors target both the priming and effector phases of T-cell activation, amplifying immune responses that increase the likelihood of irAEs. Conversely, PD-1 inhibitors such as pembrolizumab have a more limited and localized effect on T-cell activation, potentially explaining the lower incidence and severity of irAEs in patients treated with PD1 inhibitors [44].

One of the unique findings of this analysis is the observed relationship between the dose of pembrolizumab and the incidence of immune-related adverse events (irAEs). Unlike chemotherapy [45], most studies do not suggest a clear correlation between the dose level of ant-PD1 agents and the incidence of irAEs [46]. A model-based meta-analysis investigating the relationship between the incidence of irAEs and the dose/exposure of ICIs provides intriguing data in this context [46]. This meta-analysis demonstrated that PD-1 inhibitor monotherapy did not have any correlation between dose/exposure and irAEs. Intriguingly, this study also reported that a significant AE dose/exposure dependence exists for CTLA-4 inhibitor monotherapy, CTLA-4 inhibitor + PD-1 inhibitor combination therapy, and ICI + chemotherapy combination therapies for multiple AE types. Furthermore, immunotherapy-naïve patients receiving treatment with ICIs had higher AE rates than patients receiving second-line or later-line ICI therapy. Tumor characteristics, such as PD-L1 status, did not influence the observed relationships between AE rates and ICI dose/exposure. A higher incidence of irAEs with a 10 mg/kg dose every two weeks has not been previously demonstrated in studies investigating the risk factors for pembrolizumab-related irAEs [46,47]. However, a single-center retrospective analysis did report an association between the cumulative dose of pembrolizumab and the incidence of irAEs [47]. Another meta-analysis of clinical trials published between 2005 and 2018 identified a dose/exposure dependence of irAEs with CTLA-4 inhibitor monotherapy but not with PD-1 inhibitor monotherapy [46]. It is noteworthy that a phase 1 study evaluating three different dose levels of pembrolizumab (1, 3, and 10 mg/kg) found consistent toxicity across all dose levels [48,49]. Furthermore, several studies have reported no significant difference in toxicity profiles between standard dosing (200 mg every 3 weeks) and extended dosing (400 mg every 6 weeks) with pembrolizumab [50,51].

The observed higher incidence of immune-related adverse events (irAEs) with the 10 mg/kg dose of pembrolizumab every two weeks in our analysis raises important questions about dose-dependent toxicity mechanisms that warrant further investigation. While previous studies, as outlined above, including phase I trials, have generally reported consistent toxicity profiles across various pembrolizumab dosing regimens, the specific factors contributing to this finding remain unclear. Our analysis encompassed a heterogeneous cohort of gastrointestinal (GI) cancer patients, which introduces variability in patient and tumor characteristics, the immune microenvironment, and prior treatments, all of which could influence the risk of irAEs. Importantly, a multivariable analysis accounting for these potential confounders could not be conducted because of a lack of individual-level patient data, limiting our ability to draw definitive conclusions. The lack of such an analysis underscores the need for prospective studies with more homogeneous patient populations and robust statistical approaches to validate and elucidate the underlying reasons for this apparent dose-dependent effect. Such studies could provide critical insights into optimizing pembrolizumab dosing strategies while minimizing toxicity.

The safety of pembrolizumab monotherapy in a large cohort of patients with solid tumors was recently reported [16]. This pooled analysis evaluated the safety of pembrolizumab across 31 clinical trials involving 8937 patients with 19 advanced cancer types. Pembrolizumab, administered in various dosing regimens, was associated with any-grade AEs in 96.6% of patients, with grade 3–5 AEs in 50.6% and discontinuation due to AEs in 12.7%. irAEs and infusion reactions occurred in 23.7% of patients, with grade 3–5 events in 6.3%, leading to treatment discontinuation in 3.6%. Notably, the incidence of death secondary to irAEs was extremely low at 0.2%. The median onset of irAEs was 85 days. Most cases were managed with corticosteroids, including high-dose prednisone in 22.3%. These findings are consistent with the results of our study, suggesting that the safety profile of pembrolizumab monotherapy in patients with gastrointestinal cancers is comparable to that observed in the broader advanced solid tumor population.

Meta-analyses and indirect comparisons provide valuable insights into the safety profiles of pembrolizumab monotherapy versus the dual ICI combination nivolumab plus ipilimumab, highlighting significant differences in their toxicity rates and severity [17,52,53]. When comparing the toxicity profiles of pembrolizumab monotherapy versus the combination of nivolumab and ipilimumab, the nivolumab–ipilimumab combination generally exhibits a significantly higher rate of severe AEs compared to pembrolizumab monotherapy. For instance, the pivotal CheckMate-067 trial [54], which investigated the efficacy and safety of nivolumab and ipilimumab combination in patients with melanoma, reported grade 3 or higher immune-related adverse events (irAEs) in approximately 59% of patients, with nearly 36% requiring treatment discontinuation due to these toxicities. The irAEs associated with the combination therapy included colitis, hepatitis, pneumonitis, and endocrinopathies such as hypophysitis, which occur at substantially higher rates compared to those seen with PD-1 inhibitors alone. In contrast, pembrolizumab monotherapy has demonstrated a more favorable safety profile, with large-scale analyses and pivotal trials such as KEYNOTE-024 [55] and KEYNOTE-059 [27] reporting grade 3 or higher irAEs in only 9–12% of patients. Furthermore, treatment discontinuation due to irAEs occurs in approximately 6–8% of pembrolizumab-treated patients, with treatment-related mortality being exceptionally rare (<1%). This stark discrepancy in toxicity profiles is largely attributable to the distinct mechanisms of action of these therapies. Pembrolizumab, as a PD-1 inhibitor, primarily acts within the tumor microenvironment to reinvigorate exhausted T-cells and restore localized immune responses without broadly disrupting immune homeostasis [1]. This localized effect likely contributes to the relatively low incidence of systemic irAEs. In contrast, the combination of nivolumab and ipilimumab targets both PD-1 and CTLA-4 pathways, amplifying T-cell activation during both the priming and effector phases of the immune response [1]. While this dual mechanism of action enhances anti-tumor immunity and produces robust clinical responses in certain cancers, it also results in widespread immune activation, which significantly increases the risk of systemic toxicities. The addition of ipilimumab intensifies immune activation by blocking early T-cell regulatory checkpoints, leading to a more generalized immune response that is often unrestrained. Consequently, patients receiving the combination therapy are more likely to experience severe and multi-organ irAEs, necessitating rigorous monitoring and, in many cases, the use of high-dose corticosteroids or other immunosuppressive agents to manage these toxicities. Despite the higher toxicity burden, the nivolumab–ipilimumab combination may offer enhanced efficacy in select populations, particularly those with dMMR/MSI-H tumors [56], necessitating a careful risk–benefit assessment in clinical decision-making. Overall, while pembrolizumab monotherapy offers a more manageable safety profile suitable for a broader range of patients, the nivolumab–ipilimumab combination may be reserved for those who can tolerate higher toxicity risks in exchange for potentially superior clinical outcomes in specific settings. These distinctions underscore the importance of individualized therapy based on patient characteristics, tumor biology, and the anticipated benefit–risk ratio. Future research into biomarkers predicting toxicity and therapeutic response may further refine the selection of these immunotherapeutic strategies.

Several limitations of our study warrant consideration when interpreting the findings. First, the inclusion of a heterogeneous group of GI cancer patients introduced variability in baseline characteristics, tumor biology, and treatment characteristics. These factors may influence the incidence and severity of immune-related adverse events (irAEs), yet our analysis aggregated data across this diverse population to provide a unified safety profile. This approach, while necessary for practical reasons, may mask subgroup-specific differences in toxicity rates and patterns. Second, this study included a relatively modest number of patients compared to the overall population of pembrolizumab-treated individuals with GI cancers. This limitation was partly due to the decision to restrict the analysis to full-text articles, which, although ensuring data transparency and accessibility, may have excluded relevant studies available only in abstract form or unpublished data. Third, our analysis was constrained by the lack of long-term safety data, as the majority of clinical trials included in our analysis did not report late-onset toxicities. Long-term irAEs and their management are critical for understanding the full safety profile of pembrolizumab, particularly given the potential for delayed autoimmune effects. Fourth, our analysis does not include other anti-PD1 agents like single-agent nivolumab or cemiplimab. We have not included nivolumab or cemiplimab since robust data do not exist investigating the efficacy and safety of those agents in GI cancer patients. These limitations highlight the need for larger prospective studies with comprehensive patient characterization and extended follow-up to refine our understanding of pembrolizumab’s safety.

## 5. Conclusions

In conclusion, pembrolizumab monotherapy exhibits a favorable safety profile in patients with gastrointestinal cancers, with a low incidence of severe toxicities, treatment discontinuations, and mortality due to irAEs. The rarity of these severe outcomes underscores the manageable nature of pembrolizumab-associated toxicities when appropriate monitoring and timely intervention strategies are employed. This safety profile, consistent with findings from broader cancer populations, reinforces the suitability of pembrolizumab as a therapeutic option for GI cancer patients. Future studies focusing on long-term outcomes and patient-specific factors will further enhance our understanding of its safety and optimize its clinical use.

## Figures and Tables

**Table 1:** Selected full-text articles published in PubMed reporting the safety profile of pembrolizumab monotherapy in patients with GI malignancies.

Study/Author, Year	Study Phase	n	Tumor Type	Dose	Line of Treatment
Le et al., 2015 [23]	1	41	32 patients with Colorectal, 9 patients with non-colorectal cancer	10 mg/kg q 2 wks	≥2
Muro et al., 2016 [12]	1b	39	Gastric cancer	10 mg/kg q 2 wks	1
O’Neil et al., 2017 [24]	1b	23	Colorectal	10 mg/kg q 2 wks	≥2
Ott et al., 2017 [25]	1b	25	Anal Carcinoma	10 mg/kg q 2 wks	2
Doi et al., 2018 [26]	1b	23	Esophageal Carcinoma	10 mg/kg q 2 wks	3
Fuchs et al., 2018 [27]	2	259	Gastric/GEJ Cancer	200 mg q 3 wks	2
Bang et al., 2019 [28]	2	31	Gastric/GEJ Cancer	200 mg q 3 wks	1
Shah et al., 2019 [29]	2	121	Esophageal Carcinoma	200 mg q 3 wks	3
Andre et al., 2020 [3]	3	153	Colorectal	200 mg q 3 wks	1
Finn et al., 2020 [30]	3	278	HCC	200 mg q 3 wks	2
Kojima et al., 2020 [31]	3	314	Esophageal Carcinoma	200 mg q 3 wks	2
Le et al., 2020 [8]	2	124	Colorectal	200 mg q 3 wks	2
Marabelle et al., 2020 [10]	2	233	Non-colorectal (27 tumor types were represented, with endometrial, gastric, cholangiocarcinoma, and pancreatic cancers)	200 mg q 3 wks	2
Mehnert et al., 2020 [32]	1	41	Carcinoid/pancreatic NET	10 mg/kg q 2 wks	2
Shitara et al., 2020 [33]	3	256	Gastric/GEJ Cancer	200 mg q 3 wks	1
Strosberg et al., 2020 [34]	2	107	NETs of the lung, appendix, small intestine, colon, rectum, or pancreas	200 mg q 3 wks	2
Chung et al., 2021 [35]	3	47	Gastric/GEJ cancer	200 mg q 3 wks	2
De Klerk et al., 2021 [36]	2	49	Esophageal carcinoma	200 mg q 3 wks	2
Fuchs et al., 2021 [37]	3	296	Gastric/GEJ cancer	200 mg q 3 wks	2
Pedersen et al., 2021 [38]	2	40	Small-bowel adenocarcinoma	200 mg q 3 wks	2
Kudo et al., 2022 [39]	2	104	HCC	200 mg q 3 wks	2
Marabelle et al., 2022 [40]	2	112	Anal carcinoma	200 mg q 3 wks	3
Verset et al., 2022 [41]	2	51	HCC	200 mg q 3 wks	1
Ludford et al., 2023 [9]	2	35	Twenty-seven patients with colorectal cancer and eight patients with non-colorectal cancer	200 mg q 3 wks	1
Qin et. al, 2023 [42]	3	299	HCC	200 mg q 3 wks	2

Abbreviations: GEJ, gastroesophageal junction; HCC, hepatocellular carcinoma; NET, neuroendocrine tumor; q, every; wks, weeks.

**Table 2:** Summary of patient and tumor characteristics.

Characteristic	Patients with GI Malignancy Receiving Pembrolizumab Monotherapy (n = 3101)
Age, median (range), years	62 (53–68)
Sex, No. (%)	
Male	2056 (66.3)
Female	938 (30.2)
Other, not reported	107 (3.5)
ECOG performance status	
0	1313 (42.3)
1	1644 (53)
2	4 (0.2)
Other, not reported	141 (4.5)
Primary tumor location, No. (%)	
Colorectal	359 (11.6)
Hepatocellular	732 (23.6)
Esophagogastric	1435 (46.3)
Other	575 (18.5)

Abbreviations: GI, gastrointestinal; No., number; ECOG, Eastern Cooperative Oncology Group.

**Table 3:** Adverse events by organ system.

Adverse Event by Organ System	N (Any Grade Events)	Any Grade (%)	N (Grade 3/4 Events)	Grade 3/4 (%)
**Gastrointestinal**				
Diarrhea	341	11.00%	35	1.13%
Anorexia	243	7.84%	16	0.52% 0.52%
Nausea	231	7.45%	20	0.65%
AST increase	183	5.91%	70	2.26%
ALT increase	151	4.87%	40	1.29%
Hyperbilirubinemia	97	3.13%	33	1.06%
Constipation	75	2.42%	2	0.06%
Vomiting	69	2.23%	4	0.13%
Colitis	56	1.81%	21	0.68%
Hepatitis	41	1.32%	27	0.87%
Pancreatitis	16	0.52%	5	0.16%
**Dermatologic**				
Pruritus	278	8.97%	4	0.13%
Rash	207	6.68%	12	0.39%
**Musculoskeletal**				
Arthralgia	144	4.65%	6	0.19%
Myalgia	26	0.84%	3	0.10%
Myositis	14	0.45%	3	0.10%
**Endocrine**				
Hypothyroidism	279	9.00%	4	0.13%
Hyperthyroidism	126	4.07%	1	0.03%
Adrenal insufficiency	18	0.58%	7	0.23%
Hypophysitis	13	0.42%	4	0.13%
Thyroiditis	13	0.42%	2	0.06%
Type 1 DM	9	0.29%	5	0.16%
Hyperglycemia	9	0.29%	3	0.10%
**Generalized**				
Fatigue	497	16.04%	48	1.55%
Asthenia	146	4.71%	17	0.55%
Infusion reaction	28	0.90%	0	0.00%
**Renal**				
Nephritis	10	0.32%	2	0.06%
**Pulmonary**				
Pneumonitis	110	3.55%	28	0.90%
**Cardiovascular**				
Myocarditis	2	0.06%	1	0.03%

Abbreviations: N, number; ALT, alanine transaminase; AST, aspartate transaminase; DM, diabetes mellitus.

## Data Availability

No new data were created or analyzed in this study. Data sharing is not applicable to this article.

## Supplementary Materials

The following supporting information can be downloaded at https://www.mdpi.com/article/10.3390/biomedicines13010229/s1, Table S1. Search strategy.

## References

[1] Walsh R.J., Sundar R., Lim J.S.J. (2023). Immune checkpoint inhibitor combinations—current and emerging strategies. Brit. J. Cancer.

[2] Khoja L., Butler M.O., Kang S.P., Ebbinghaus S., Joshua A.M. (2015). Pembrolizumab. J. ImmunoTherapy Cancer.

[3] Andre T., Shiu K.K., Kim T.W., Jensen B.V., Jensen L.H., Punt C., Smith D., Garcia-Carbonero R., Benavides M., Gibbs P. (2020). Pembrolizumab in Microsatellite-Instability-High Advanced Colorectal Cancer. N. Engl. J. Med..

[4] Chakrabarti S., Parish M., Peterson C., Ludwig K.A., Sriram D., Ruggeri A., Tolay S., Selfridge J.E., Bajor D.L., Mohamed A. (2023). The efficacy and safety of neoadjuvant immunotherapy in patients with deficient mismatch repair/microsatellite instability–high (dMMR/MSI-H) localized and oligometastatic colon cancer: Data from the real world. J. Clin. Oncol..

[5] Diaz L.A., Shiu K.K., Kim T.W., Jensen B.V., Jensen L.H., Punt C., Smith D., Garcia-Carbonero R., Benavides M., Gibbs P. (2022). Pembrolizumab versus chemotherapy for microsatellite instability-high or mismatch repair-deficient metastatic colorectal cancer (KEYNOTE-177): Final analysis of a randomised, open-label, phase 3 study. Lancet Oncol..

[6] Eefsen R.L., Larsen J.S., Klarskov L.L., Altaf R., Høgdall E., Ingeholm P., Lykke J., Nielsen D.L., Pfeiffer P., Poulsen L. (2023). Therapy with pembrolizumab in treatment-naïve patients with nonmetastatic, mismatch repair deficient colorectal cancer. Int. J. Cancer.

[7] Janjigian Y.Y., Kawazoe A., Bai Y., Xu J., Lonardi S., Metges J.P., Yanez P., Wyrwicz L.S., Shen L., Ostapenko Y. (2023). Pembrolizumab plus trastuzumab and chemotherapy for HER2-positive gastric or gastro-oesophageal junction adenocarcinoma: Interim analyses from the phase 3 KEYNOTE-811 randomised placebo-controlled trial. Lancet.

[8] Le D.T., Kim T.W., Cutsem E.V., Geva R., Jäger D., Hara H., Burge M., O’neil B., Kavan P., Yoshino T. (2020). Phase II Open-Label Study of Pembrolizumab in Treatment-Refractory, Microsatellite Instability–High/Mismatch Repair–Deficient Metastatic Colorectal Cancer: KEYNOTE-164. J. Clin. Oncol..

[9] Ludford K., Ho W.J., Thomas J.V., Raghav K.P., Murphy M.B., Fleming N.D., Lee M.S., Smaglo B.G., You Y.N., Tillman M.M. (2023). Neoadjuvant Pembrolizumab in Localized Microsatellite Instability High/Deficient Mismatch Repair Solid Tumors. J. Clin. Oncol..

[10] Marabelle A., Le D.T., Ascierto P.A., Di Giacomo A.M., De Jesus-Acosta A., Delord J.-P., Geva R., Gottfried M., Penel N., Hansen A.R. (2020). Efficacy of Pembrolizumab in Patients With Noncolorectal High Microsatellite Instability/Mismatch Repair-Deficient Cancer: Results From the Phase II KEYNOTE-158 Study. J. Clin. Oncol..

[11] Marcus L., Lemery S.J., Keegan P., Pazdur R. (2019). FDA Approval Summary: Pembrolizumab for the treatment of microsatellite instability-high solid tumors. Clin. Cancer Res..

[12] Muro K., Chung H.C., Shankaran V., Geva R., Catenacci D., Gupta S., Eder J.P., Golan T., Le D.T., Burtness B. (2016). Pembrolizumab for patients with PD-L1-positive advanced gastric cancer (KEYNOTE-012): A multicentre, open-label, phase 1b trial. Lancet Oncol..

[13] Rha S.Y., Oh D.-Y., Yañez P., Bai Y., Ryu M.-H., Lee J., Rivera F., Alves G.V., Garrido M., Shiu K.-K. (2023). Pembrolizumab plus chemotherapy versus placebo plus chemotherapy for HER2-negative advanced gastric cancer (KEYNOTE-859): A multicentre, randomised, double-blind, phase 3 trial. Lancet Oncol..

[14] Sun J.-M., Shen L., Shah M.A., Enzinger P., Adenis A., Doi T., Kojima T., Metges J.-P., Li Z., Kim S.-B. (2021). Pembrolizumab plus chemotherapy versus chemotherapy alone for first-line treatment of advanced oesophageal cancer (KEYNOTE-590): A randomised, placebo-controlled, phase 3 study. Lancet.

[15] Kennedy L.B., Salama A.K.S. (2020). A review of cancer immunotherapy toxicity. CA Cancer J. Clin..

[16] Brahmer J.R., Long G.V., Hamid O., Garon E.B., Herbst R.S., Andre T., Armand P., Bajorin D., Bellmunt J., Burtness B. (2024). Safety profile of pembrolizumab monotherapy based on an aggregate safety evaluation of 8937 patients. Eur. J. Cancer.

[17] Almutairi A.R., McBride A., Slack M., Erstad B.L., Abraham I. (2020). Potential Immune-Related Adverse Events Associated With Monotherapy and Combination Therapy of Ipilimumab, Nivolumab, and Pembrolizumab for Advanced Melanoma: A Systematic Review and Meta-Analysis. Front. Oncol..

[18] Nishino M., Giobbie-Hurder A., Hatabu H., Ramaiya N.H., Hodi F.S. (2016). Incidence of Programmed Cell Death 1 Inhibitor-Related Pneumonitis in Patients With Advanced Cancer: A Systematic Review and Meta-analysis. JAMA Oncol..

[19] Sher A.F., Golshani G.M., Wu S. (2020). Fatal Adverse Events Associated with Pembrolizumab in Cancer Patients: A Meta-Analysis. Cancer Investig..

[20] Udayakumar S., Parmar A., Leighl N.B., Everest L., Arciero V.S., Santos S.D., Rahmadian A., Doherty M.K., Chan K.K.W. (2022). Pembrolizumab alone or with chemotherapy for metastatic non-small-cell lung cancer: A systematic review and network meta-analysis. Crit. Rev. Oncol./Hematol..

[21] Wang W., Lie P., Guo M., He J. (2017). Risk of hepatotoxicity in cancer patients treated with immune checkpoint inhibitors: A systematic review and meta-analysis of published data. Int. J. Cancer.

[22] Zhou X., Yao Z., Bai H., Duan J., Wang Z., Wang X., Zhang X., Xu J., Fei K., Zhang Z. (2021). Treatment-related adverse events of PD-1 and PD-L1 inhibitor-based combination therapies in clinical trials: A systematic review and meta-analysis. Lancet Oncol..

[23] Le D.T., Uram J.N., Wang H., Bartlett B.R., Kemberling H., Eyring A.D., Skora A.D., Luber B.S., Azad N.S., Laheru D. (2015). PD-1 Blockade in Tumors with Mismatch-Repair Deficiency. N. Engl. J. Med..

[24] O’Neil B.H., Wallmark J.M., Lorente D., Elez E., Raimbourg J., Gomez-Roca C., Ejadi S., Piha-Paul S.A., Stein M.N., Razak A.R.A. (2017). Safety and antitumor activity of the anti–PD-1 antibody pembrolizumab in patients with advanced colorectal carcinoma. PLoS ONE.

[25] Ott P.A., Piha-Paul S.A., Munster P., Pishvaian M.J., van Brummelen E.M.J., Cohen R.B., Gomez-Roca C., Ejadi S., Stein M., Chan E. (2017). Safety and antitumor activity of the anti-PD-1 antibody pembrolizumab in patients with recurrent carcinoma of the anal canal. Ann. Oncol..

[26] Doi T., Piha-Paul S.A., Jalal S.I., Saraf S., Lunceford J., Koshiji M., Bennouna J. (2018). Safety and Antitumor Activity of the Anti–Programmed Death-1 Antibody Pembrolizumab in Patients With Advanced Esophageal Carcinoma. J. Clin. Oncol..

[27] Fuchs C.S., Doi T., Jang R.W., Muro K., Satoh T., Machado M., Sun W., Jalal S.I., Shah M.A., Metges J.-P. (2018). Safety and Efficacy of Pembrolizumab Monotherapy in Patients with Previously Treated Advanced Gastric and Gastroesophageal Junction Cancer. JAMA Oncol..

[28] Bang Y.-J., Kang Y.-K., Catenacci D.V., Muro K., Fuchs C.S., Geva R., Hara H., Golan T., Garrido M., Jalal S.I. (2019). Pembrolizumab alone or in combination with chemotherapy as first-line therapy for patients with advanced gastric or gastroesophageal junction adenocarcinoma: Results from the phase II nonrandomized KEYNOTE-059 study. Gastric Cancer.

[29] Shah M.A., Kojima T., Hochhauser D., Enzinger P., Raimbourg J., Hollebecque A., Lordick F., Kim S.-B., Tajika M., Kim H.T. (2019). Efficacy and Safety of Pembrolizumab for Heavily Pretreated Patients With Advanced, Metastatic Adenocarcinoma or Squamous Cell Carcinoma of the Esophagus. JAMA Oncol..

[30] Finn R.S., Ryoo B.-Y., Merle P., Kudo M., Bouattour M., Lim H.Y., Breder V., Edeline J., Chao Y., Ogasawara S. (2020). Pembrolizumab As Second-Line Therapy in Patients With Advanced Hepatocellular Carcinoma in KEYNOTE-240: A Randomized, Double-Blind, Phase III Trial. J. Clin. Oncol..

[31] Kojima T., Shah M.A., Muro K., Francois E., Adenis A., Hsu C.-H., Doi T., Moriwaki T., Kim S.-B., Lee S.-H. (2020). Randomized Phase III KEYNOTE-181 Study of Pembrolizumab Versus Chemotherapy in Advanced Esophageal Cancer. J. Clin. Oncol..

[32] Mehnert J.M., Bergsland E., O’Neil B.H., Santoro A., Schellens J.H.M., Cohen R.B., Doi T., Ott P.A., Pishvaian M.J., Puzanov I. (2020). Pembrolizumab for the treatment of programmed death-ligand 1-positive advanced carcinoid or pancreatic neuroendocrine tumors: Results from the KEYNOTE-028 study. Cancer.

[33] Shitara K., Van Cutsem E., Bang Y.-J., Fuchs C., Wyrwicz L., Lee K.-W., Kudaba I., Garrido M., Chung H.C., Lee J. (2020). Efficacy and Safety of Pembrolizumab or Pembrolizumab Plus Chemotherapy vs Chemotherapy Alone for Patients With First-line, Advanced Gastric Cancer. JAMA Oncol..

[34] Strosberg J.R., Mizuno N., Doi T., Grande E., Delord J.-P., Shapira-Frommer R., Bergsland E.K., Shah M.H., Fakih M., Takahashi S. (2020). Efficacy and Safety of Pembrolizumab in Previously Treated Advanced Neuroendocrine Tumors: Results From the Phase II KEYNOTE-158 Study. Clin. Cancer Res..

[35] Chung H.C., Kang Y.K., Chen Z., Bai Y., Ishak W.Z.W., Shim B.Y., Park Y.L., Koo D., Lu J., Xu J. (2021). Pembrolizumab versus paclitaxel for previously treated advanced gastric or gastroesophageal junction cancer (KEYNOTE-063): A randomized, open-label, phase 3 trial in Asian patients. Cancer.

[36] de Klerk L.K., Patel A.K., Derks S., Pectasides E., Augustin J., Uduman M., Raman N., Akarca F.G., McCleary N.J., Cleary J.M. (2021). Phase II study of pembrolizumab in refractory esophageal cancer with correlates of response and survival. J. ImmunoTherapy Cancer.

[37] Fuchs C.S., Özgüroğlu M., Bang Y.-J., Di Bartolomeo M., Mandala M., Ryu M.-H., Fornaro L., Olesinski T., Caglevic C., Chung H.C. (2021). Pembrolizumab versus paclitaxel for previously treated PD-L1-positive advanced gastric or gastroesophageal junction cancer: 2-year update of the randomized phase 3 KEYNOTE-061 trial. Gastric Cancer.

[38] Pedersen K.S., Foster N.R., Overman M.J., Boland P.M., Kim S.S., Arrambide K.A., Jaszewski B.L., Bekaii-Saab T., Graham R.P., Welch J. (2021). ZEBRA: A Multicenter Phase II Study of Pembrolizumab in Patients with Advanced Small-Bowel Adenocarcinoma. Clin. Cancer Res..

[39] Kudo M., Finn R.S., Edeline J., Cattan S., Ogasawara S., Palmer D.H., Verslype C., Zagonel V., Fartoux L., Vogel A. (2022). Updated efficacy and safety of KEYNOTE-224: A phase II study of pembrolizumab in patients with advanced hepatocellular carcinoma previously treated with sorafenib. Eur. J. Cancer.

[40] Marabelle A., Cassier P.A., Fakih M., Kao S., Nielsen D., Italiano A., Guren T.K., van Dongen M.G.J., Spencer K., Bariani G.M. (2022). Pembrolizumab for previously treated advanced anal squamous cell carcinoma: Results from the non-randomised, multicohort, multicentre, phase 2 KEYNOTE-158 study. Lancet Gastroenterol. Hepatol..

[41] Verset G., Borbath I., Karwal M., Verslype C., Van Vlierberghe H., Kardosh A., Zagonel V., Stal P., Sarker D., Palmer D.H. (2022). Pembrolizumab Monotherapy for Previously Untreated Advanced Hepatocellular Carcinoma: Data from the Open-Label, Phase II KEYNOTE-224 Trial. Clin. Cancer Res..

[42] Qin S., Chen Z., Fang W., Ren Z., Xu R., Ryoo B.-Y., Meng Z., Bai Y., Chen X., Liu X. (2023). Pembrolizumab Versus Placebo as Second-Line Therapy in Patients From Asia With Advanced Hepatocellular Carcinoma: A Randomized, Double-Blind, Phase III Trial. J. Clin. Oncol..

[43] Bakacs T., Moss R.W., Kleef R., Szasz M.A., Anderson C.C. (2019). Exploiting autoimmunity unleashed by low-dose immune checkpoint blockade to treat advanced cancer. Scand. J. Immunol..

[44] Francisco L.M., Sage P.T., Sharpe A.H. (2010). The PD-1 pathway in tolerance and autoimmunity. Immunol. Rev..

[45] Gurney H. (2002). How to calculate the dose of chemotherapy. Br. J. Cancer.

[46] Shulgin B., Kosinsky Y., Omelchenko A., Chu L., Mugundu G., Aksenov S., Pimentel R., DeYulia G., Kim G., Peskov K. (2020). Dose dependence of treatment-related adverse events for immune checkpoint inhibitor therapies: A model-based meta-analysis. OncoImmunology.

[47] Eun Y., Kim I.Y., Sun J.-M., Lee J., Cha H.-S., Koh E.-M., Kim H., Lee J. (2019). Risk factors for immune-related adverse events associated with anti-PD-1 pembrolizumab. Sci. Rep..

[48] Garon E.B., Rizvi N.A., Hui R., Leighl N., Balmanoukian A.S., Eder J.P., Patnaik A., Aggarwal C., Gubens M., Horn L. (2015). Pembrolizumab for the Treatment of Non–Small-Cell Lung Cancer. N. Engl. J. Med..

[49] Renner A., Burotto M., Rojas C. (2019). Immune Checkpoint Inhibitor Dosing: Can We Go Lower Without Compromising Clinical Efficacy?. J. Glob. Oncol..

[50] Dubé-Pelletier M., Labbé C., Côté J., Pelletier-St-Pierre A.-A. (2023). Pembrolizumab Every 6 Weeks Versus Every 3 Weeks in Advanced Non-Small Cell Lung Cancer. Oncologist.

[51] Higashiyama R.I., Yoshida T., Yagishita S., Ohuchi M., Sakiyama N., Torasawa M., Shirasawa M., Masuda K., Shinno Y., Matsumoto Y. (2022). Safety Implications of Switching Pembrolizumab Dosage From 200 mg Every 3 Weeks to 400 mg Every 6 Weeks in Patients With Advanced NSCLC. J. Thorac. Oncol..

[52] O’Byrne K., Popoff E., Badin F., Lee A., Yuan Y., Lozano-Ortega G., Eccles L.J., Varol N., Waser N., Penrod J.R. (2023). Long-term comparative efficacy and safety of nivolumab plus ipilimumab relative to other first-line therapies for advanced non-small-cell lung cancer: A systematic literature review and network meta-analysis. Lung Cancer.

[53] Zhou Y., Zhang Y., Guo G., Cai X., Yu H., Cai Y., Zhang B., Hong S., Zhang L. (2020). Nivolumab plus ipilimumab versus pembrolizumab as chemotherapy-free, first-line treatment for PD-L1-positive non-small cell lung cancer. Clin. Transl. Med..

[54] Wolchok J.D., Chiarion-Sileni V., Gonzalez R., Rutkowski P., Grob J.-J., Cowey C.L., Lao C.D., Wagstaff J., Schadendorf D., Ferrucci P.F. (2017). Overall Survival with Combined Nivolumab and Ipilimumab in Advanced Melanoma. N. Engl. J. Med..

[55] Reck M., Rodríguez-Abreu D., Robinson A.G., Hui R., Csőszi T., Fülöp A., Gottfried M., Peled N., Tafreshi A., Cuffe S. (2016). Pembrolizumab versus Chemotherapy for PD-L1–Positive Non–Small-Cell Lung Cancer. N. Engl. J. Med..

[56] Lenz H.J., Van Cutsem E., Luisa Limon M., Wong K.Y.M., Hendlisz A., Aglietta M., García-Alfonso P., Neyns B., Luppi G., Cardin D.B. (2022). First-Line Nivolumab Plus Low-Dose Ipilimumab for Microsatellite Instability-High/Mismatch Repair-Deficient Metastatic Colorectal Cancer: The Phase II CheckMate 142 Study. J. Clin. Oncol..

