# Supplementary material for: Toxicity Associated with Pembrolizumab Monotherapy in Patients with Gastrointestinal Cancers: A Systematic Review of Clinical Trials
Source: Biomedicines. 2025 Jan 18;13(1):229. doi: 10.3390/biomedicines13010229 (PMC11762711; doi:10.3390/biomedicines13010229)
Supplement: Supplementary file 1 [file biomedicines-13-00229-s001.zip › biomedicines-3415302-supplementary.pdf]

Table S1. Search Strategy

Database	Date Accessed	Results	Duplicates	Search Strategy
Pubmed	24 April 2024	118	0	"pembrolizumab"[Title/Abstract] AND ("colonic neoplasms"[MeSH Terms] OR "rectal neoplasms"[MeSH Terms] OR "colorectal neoplasms"[MeSH Terms] OR "stomach neoplasms"[MeSH Terms] OR "esophageal neoplasms"[MeSH Terms] OR "esophagogastric junction"[MeSH Terms] OR "carcinoma, hepatocellular"[MeSH Terms] OR "biliary tract neoplasms"[MeSH Terms] OR "cholangiocarcinoma"[MeSH Terms] OR "gallbladder neoplasms"[MeSH Terms] OR "intestinal neoplasms"[MeSH Terms] OR "pancreatic neoplasms"[MeSH Terms] OR (("anal canal"[MeSH Terms] OR ("Anal"[All Fields] AND "canal"[All Fields]) OR "anal canal"[All Fields]) AND "neoplasms"[MeSH Terms]) OR ("Anal"[All Fields] AND "carcinoma, squamous cell"[MeSH Terms]) OR (("neuroendocrin"[All Fields] OR "neuroendocrinal"[All Fields] OR "neuroendocrines"[All Fields] OR "neurosecretory systems"[MeSH Terms] OR ("neurosecretory"[All Fields] AND "systems"[All Fields]) OR "neurosecretory systems"[All Fields] OR "neuroendocrine"[All Fields]) AND "neoplasms"[MeSH Terms])) AND "clinical trial"[Publication Type]
